# Tumour necrosis factor allele variants and their association with the occurrence and severity of malaria in African children: a longitudinal study

**DOI:** 10.1186/s12936-015-0767-3

**Published:** 2015-06-20

**Authors:** Wanjiku N Gichohi-Wainaina, Alida Melse-Boonstra, Edith J Feskens, Ayse Y Demir, Jacobien Veenemans, Hans Verhoef

**Affiliations:** Division of Human Nutrition, Wageningen University, Wageningen, The Netherlands; Laboratory for Clinical Chemistry, Meander Medical Centre, Amersfoort, The Netherlands; Laboratory for Microbiology and Infection Control, Amphia Hospital, Breda, The Netherlands; Cell Biology and Immunology Group, Wageningen University, Wageningen, The Netherlands; Medical Research Council (MRC) International Nutrition Group, London School of Hygiene & Tropical Medicine, London, UK; Medical Research Council (MRC), Keneba, The Gambia

**Keywords:** Tumour necrosis factor, Malaria, *Plasmodium* infection

## Abstract

**Background:**

Tumour necrosis factor (*TNF*) is central to the immune response to *Plasmodium* infection. Its plasma concentration is influenced by allele variants in the promoter region of *TNF*. The study’s objectives were to assess *TNF* allele variants (*TNF*_−1031_, *TNF*_−308_): (1) modulation of malaria rates in young Tanzanian children; (2) modulation of the severity of malaria as indicated by haemoglobin concentrations at the time of presentation with febrile episodes; and (3) the association between *Plasmodium* infection and haemoglobin concentration in symptomless parasite carriers.

**Methods:**

Data from a placebo-controlled trial in which 612 Tanzanian children aged 6–60 months with height-for-age z-score in the range −3 SD to 1.5 SD was utilised. Those with *Plasmodium* infection at baseline were treated with artemether-lumefantrine. An episode of malaria was predefined as current *Plasmodium* infection with an inflammatory response (axillary temperature ≥37.5°C or whole blood C-reactive protein concentration ≥8 mg/L) in children reported sick. Linkage disequilibrium (LD) pattern assessment as well as haplotype analysis was conducted using HAPLOVIEW. Cox regression models used in the primary analysis accounted for multiple episodes per child.

**Results:**

Genotyping of 94.9% (581/612) children for *TNF*_−1031_ (*TNF*_−1031_T>C); allele frequency was 0.39. Corresponding values for rs1800629 (*TNF*_−308_G>A) were 95.4% (584/612) and 0.17. Compared to the wild type genotype (TT), malaria rates were increased in the *TNF*_−1031_CC genotype (hazard ratio, HR [95% CI]: 1.41 [1.01‒1.97] and 1.31 [0.97‒1.76] for crude analysis and adjusting for pre-specified baseline factors, respectively) but decreased in those with the *TNF*_−308_AA genotype (corresponding HR: 0.13 [0.02‒0.63] and 0.16 [0.04‒0.67]). These associations were weaker when analysing first episodes of malaria (*P* value −0.59 and 0.38, respectively). No evidence that allele variants of *TNF*_−1031_ and *TNF*_−308_ affected haemoglobin concentration at first episode of malaria, or that they modified the association between *Plasmodium* infection and haemoglobin concentrations at baseline was observed.

**Conclusion:**

In this cohort of Tanzanian children, the *TNF*_−1031_CC genotype was associated with increased rates of malarial episodes, whereas the *TNF*_−308_AA genotype was associated with decreased rates.

## Background

Tumour necrosis factor (*TNF*) is central to the immune response to *Plasmodium* infection because of its pyrogenic properties and its key role in triggering the cascade of pro-inflammatory cytokines that regulate immune cells. It is secreted predominantly by activated macrophages following exposure to *Plasmodium* antigens, and acts to suppress parasitaemia.

Eight variants have been described within the *TNF* promoter at positions 1031T>C (rs1799964), −863C>A (rs1800630), −857C>T (rs1799724), −575G>A, −376G>A (rs1800750), −308G>A (rs1800629), −244G>A, and −238G>A (rs361525) relative to the transcription start site [1–7]. These genetic variants are investigated due to the possibility that the genetic changes they introduce, in comparison to the wild type form, could affect the binding of transcription factors, thereby controlling the activity of the promoter sites and affecting resulting mRNA and protein concentrations. There are many studies showing association of *TNF* variants with various infectious diseases, but others failed to show an association [8].

In relation to malaria, the *TNF*_−*1031*_C allele has been associated with decreased risk of severe malaria in a child cohort of Gambian malaria patients but this observation was not made in similar cohorts from Kenya and Malawi [9]. Also observations from a case–control study in India indicate that the *TNF*_−*1031*_C allele is associated with elevated plasma TNF concentrations and increased susceptibility to severe falciparum malaria [10]. *TNF*_−308_AA has been associated with severe cerebral malaria in Gambian children, and with a seven-fold increased risk of death and severe cerebral malaria [11]. Furthermore, a study of Gabon school children observed the *TNF*_−308_A allele to be associated with shorter intervals of malaria reinfection [12]. A Kenyan study investigating malarial morbidity in young children found this variant to be associated with preterm birth and early childhood mortality and malaria morbidity, with carriers showing a trend towards high density parasitaemia and severe malarial anaemia [13]. Another study from Sri Lanka found that the *TNF*_−308_AA was associated with both severe malaria and other infections [14]. Some studies however have failed to observe any association between *TNF*_−308_AA and malaria. A study of malaria patients from Thailand found no association between *TNF*_−308_AA and disease severity. This finding is similar to that of Ubalee et al. [15], which did not observe a difference in severe symptoms between patients in Myanmar on the basis of *TNF*_−308_AA [15]. Another study of Tanzanian infants observed minor clinical parameters to differ in *TNF*_−308_GA heterozygotes but did not observe major differences in other malaria related indices [16].

There have been few cohort studies to longitudinally assess the association of *TNF* allele variants on malaria outcomes. Children with an elevated TNF response to *Plasmodium* infection are more likely to become sicker than their peers with a lower TNF response capacity, so that the former may experience more frequent episodes of malaria. On the other hand, in children exposed to chronic or repeated *Plasmodium* infections, an increased TNF response associated with the TNF_−308_AA genotype may intensify a hepcidin-mediated block in iron absorption and consequently induce or exacerbate iron deficiency [17], which probably protects against malarial episodes [18, 19].

The aim of this study was therefore to assess the associations of *TNF*_−1031_ and *TNF*_−308_ allele variants with malaria rates in young Tanzanian children. Additionally, the study aimed to investigate the association of these variants on the severity of malaria as indicated by haemoglobin concentrations at the time of presentation with febrile episodes. Lastly, how these variants modulated the association between *Plasmodium* infection and haemoglobin concentration in symptomless parasite carriers was assessed.

## Methods

### Study area and population

Data from a randomized trial that aimed to assess the effect of supplementation with zinc and other micronutrients on malaria rates was used. The trial was conducted between February 2008 and March 2009 in four rural villages in Handeni District, northeastern Tanzania. Residents primarily belong to the Wazigua and Wabondei Bantu tribes, but settlement of migrant plantation workers has resulted in a mixture of tribes with different origins and much intermarriage. The area is mainly populated by poor farmer families involved in subsistence farming. Malaria transmission is intense and perennial, with nearly all infections being due to *Plasmodium falciparum* [20]. Apart from several local traditional healers, the research dispensary was the only health facility in the area. The study was approved by the Ethical Review Committee of Wageningen University, The Netherlands (Approval number: NL: 04/07) and the National Health Research Ethics Review sub-Committee, Dar es Salaam, Tanzania (Approval number: NIMR/HQ/R.8a/Vol. IX/540). Informed consent was obtained from community leaders, local government officials and parents or guardians. Further details of this trial are described elsewhere [21].

### Recruitment

All resident children were eligible for randomization when aged 6–59 months and with a height-for-age z-score in the range −3 SD to 1.5 SD. Children with haemoglobin concentration <70 g/L, signs of chronic illness, and those unlikely to remain permanently resident or comply with the supplementation for the duration of the trial, or whose parents or guardians declined consent, were excluded from the study. Venous blood samples were collected in EDTA tubes and centrifuged immediately. An aliquot of 90 μL erythrocyte sediment with the buffy coat was mixed with 90 μL phosphate-buffered saline and 180 μL of DNA stabilizing buffer (AS1; Qiagen, Hilden, Germany) and stored at 4°C for subsequent genotyping. Plasma samples were stored in liquid nitrogen in the field and at −80°C during transport and subsequent storage until biochemical analysis in The Netherlands. Haemoglobin concentration was measured in an aliquot of whole blood by a haematology analyser (Sysmex KX21, Kobe, Japan). *Plasmodium* infection was detected in fresh blood by rapid dipstick test (CareStart, Access Bio, Monmouth Jct, USA). Children with a positive test result were treated immediately with artemether-lumefantrine. The location of the child’s homestead was determined using a global positioning system. Further details about recruitment procedures are reported elsewhere [21].

### Experimental intervention

Children were randomized within six strata defined by *Plasmodium* infection (binary) and age class (6–17 months, 18–35 months and 36–60 months) and randomly permuted blocks with size randomly selected of four or eight. They then received daily supplements with either zinc alone (10 mg as gluconate), multi-nutrients without zinc, zinc combined with multi-nutrients or placebo. Supplements, in the form of powder in colour-coded capsules, were contained in blister packs, and administered orally after suspending capsule contents in clean water or breastmilk. Supplementation was performed by local community volunteers, who reported adherence daily to field staff at the research dispensary.

### Follow-up and case detection

A clinical officer was on duty at the research clinic day and night. At recruitment, parents or guardians were requested to bring participating children to the dispensary immediately when detecting fever or any other illness during the intervention period. In samples collected at baseline and from sick children, the presence of parasite-specific lactate dehydrogenase (*P. falciparum* and other *Plasmodium* species) was detected by rapid tests (CareStart, G0121, Access Bio, Monmouth Jct, NJ). Axillary temperature was measured using an electronic thermometer and dipstick tests were administered for children with guardian-reported fever; for those with positive test results, plasma samples were collected and measured whole-blood C-reactive protein concentrations using a point-of-care test. Plasma was stored as described for the recruitment procedure. Artemether-lumefantrine (Novartis Pharma, Basel, Switzerland) was administered to any child with current *Plasmodium* infection upon enrolment, or with reported fever and a positive dipstick test result during the follow-up period.

### Laboratory procedures

For children who presented with malarial episodes, whole-blood concentrations of haemoglobin and C-reactive protein were measured using point-of-care tests (HemoCue, Ängelholm, Sweden and QuikRead, Orion Diagnostica, Espoo, Finland, respectively). Plasma concentrations of *P. falciparum*-specific histidine-rich protein-2 (HRP2) in samples collected during the first malaria episode were measured using a commercial enzyme-linked immunosorbent assay kit (Malaria Ag Celisa; Cellabs, Brookvale, NSW, Australia). Plasma concentrations of C-reactive protein were measured (Meander Medical Centre, Amersfoort, The Netherlands) on a Beckman Coulter Unicel DxC880i system according to the manufacturer’s instructions. Genotypes were determined using Illumina’s VeraCode™ GoldenGate Genotyping Assay on a BeadXpress™ platform. *TNF* variant allele clustering was assessed visually to determine success of genotyping. Further quality control cut-offs were: a GenCall Score of >0.5 and a call rate of ≥0.95 [22]. For the variant *TNF*_−1031,_ the reference allele was T and the alternate C. Individuals homozygous for the *TNF*_−1031_ reference allele (T) are hereon described as wild type while those homozygous for the alternate allele (C) are referred to as homozygote mutant. For the variant *TNF*_−308_, the reference allele was G and the alternate was A. Individuals homozygous for the *TNF*_−308_ reference allele (G) are hereon described as wild type while those homozygous for the alternate allele (A) are referred to as homozygote mutant.

### Statistical analyses

Linkage disequilibrium (LD) pattern assessment as well as haplotype analysis was conducted using HAPLOVIEW [23]. Anthropometric indices were calculated using Epi Info software [24] All analyses were performed using SPSS (v15.0 for Windows, SPSS, Chicago, IL, USA), CIA (v2.1.2) [25] and STATA (v11; College Station, TX, USA). For each TNF genotype, Fisher’s exact test was used to assess whether populations were in Hardy–Weinberg equilibrium. The differences in baseline characteristics were calculated using the homozygote wild-type group as the reference for each variant using CIA (v2.1.2). Normality of variables was assessed by visual inspection of histograms. Because variables of interest (age, haemoglobin concentrations, distance from homestead to dispensary and anthropometric indices) were normally distributed, means, SDs and 95% CIs were reported.

### Association of TNF genotype with malaria rates

The primary outcome, an episode of malaria, was predefined as a positive result for either a pLDH or a HRP2 dipstick test with any of the following: (a) confirmed fever (axillary temperature ≥37.5°C as measured by electronic thermometer); or, (b) guardian-reported but unconfirmed 24-h history of fever in the presence of inflammation (whole blood C-reactive protein concentrations >8 mg/L), separated by at least 14 days from a previous malaria episode. Incidence per *TNF* genotype and incidence ratios based on time to first episodes, with wild-type homozygotes as reference group was calculated. In the primary analysis, group rates were compared using Cox regression with robust estimates of the standard error to account for multiple episodes within children. The extent to which supplementation with either zinc or multi-nutrients including iron modulated the magnitude of the association of *TNF* genotype on malaria rates was explored. The extent to which adjustment for baseline factors that were a priori expected to be prognostic for malaria (*Plasmodium* infection status, distance between homestead and clinic [continuous variable], height-for-age z-score [continuous variable], mosquito net use [binary variable]) and experimental intervention modulated the estimated association of genotypes were explored. In this adjusted analysis, experimental intervention as a binary variable indicating pooled groups receiving multi-nutrients (with or without zinc) and pooled groups receiving no multi-nutrients (with or without zinc) was included. Kaplan–Meier analysis with Peto tests were used to assess associations of *TNF* genotypes with time-to-first episode of malaria.

### TNF genotype modulation of the association between *Plasmodium falciparum* infection and haemoglobin concentration at baseline

Since the additive genetic model was considered, for each genotype, two dummy variables were used to indicate the three classes, resulting in two interaction terms per variant. Regression models were then used to assess the modulation of genotypes on the associations between *P. falciparum* and haemoglobin concentration. Main terms for *P. falciparum* infection and genotype dummies were included, and adjusted as pre planned for age class, mosquito net use, height-for-age z-score, and distance between the child’s homestead and the dispensary in regression analyses.

### TNF genotype modulation of the association between *Plasmodium falciparum* infection and haemoglobin concentration at first episode of malaria

The additive genetic model was considered so for each genotype, two dummy variables were used to indicate the three classes. TNF genotype modulation of the association between *P. falciparum* infection and haemoglobin concentration at first episode of malaria was then assessed by analysis of variance (ANOVA) and adjusted for age class, height-for-age z-score and distance between the child’s homestead and the dispensary and treatment group.

## Results

Of 612 children recruited in the original study, 581 (94.9%) and 584 (95.4%) had DNA typed for *TNF*_−1031_ and *TNF*_−308_, respectively. Only 3% (20/612) of children failed to complete the trial. Minor allele frequencies were 0.39 (*TNF*_−1031_) and 0.17 (*TNF*_−308_), respectively. Neither of the *TNF* variants showed a departure from Hardy–Weinberg equilibrium (P = 0.90 and 0.57 for *TNF*_−1031_ and *TNF*_−308_, respectively). Additionally, the variants *TNF*_−1031_ is not in linkage disequilibrium with *TNF*_−308_ (r^2^ = 0.03). No haplotype blocks were identified.

### Baseline factors associated with *TNF* genotypes

Children with *TNF*_−1031_C allele seemed to have larger weight-for-age z-scores than their peers with the wild type variant (0.14 SD and 0.41 SD in heterozygotes and homozygotes, respectively, compared to −0.18 SD in those with the wild type genotype) (Table 1). The *TNF*_−308_A allele was marginally significantly associated with increased *Plasmodium* infection, but the evidence was weak (p-value: 0.09). No evidence of other factors being associated with the *TNF* genotypes investigated was observed.

**Table 1:** Factors associated with rs1799964 and rs1800629 allele variants of the *TNF* gene

Allele variant/factor	Wild type	Heterozygote	Difference (95% CI)	Homozygote mutant	Difference (95% CI)	p-value
rs1799964	−1031TT	−1031TC		−1031CC		
n	356	200		25		
Age (months)	33.4 ± 15.5	31.9 ± 16.1	−1.5 (−4.2,1.2)	28.06 ± 14.9	−5.3 (−11.6, 0.99)	0.18
Sex (%male)	48.0% (171)	48.0% (96)	0.0% (−8.6%, 8.6%)	44.0% (11)	−4.0% (−22.1%, 15.6%)	0.93
Haemoglobin concentration, g/L	102.7 ± 12.3	103.4 ± 13.8	0.0 (−2.1,2.1)	101.5 ± 10.4	−1.2 (−6.2, 3.8)	0.71
Anaemia, % (n)^a^	69.1% (246)	66.0% (132)	−3.1% (−11.3%, 4.9%)	76.0% (19)	6.9% (−13.1%, 20.4%)	0.50
*Plasmodium* infection, % (n)	43.8% (156)	43.0% (86)	−0.8% (−9.3%, 7.8%)	36.0% (9)	−7.8% (−24.4%, 12.3%)	0.75
Inflammation, % (n)^b^	32.3% (114)	32.0% (64)	0.0% (−7.9%, 8.2%)	32.0% (8)	−0.3% (−15.6%, 19.8%)	0.58
Mosquito net use, % (n)^c^	32.9% (117)	32.0% (64)	−0.1% (−8.8%, 7.3%)	32.0% (8)	−0.9% (−16.5%, 19.3%)	0.99
Distance from homestead to dispensary, km^d^	3.5 ± 2.1	3.5 ± 2.2	0.0 (−0.4, 0.4)	3.8 ± 2.6	0.3 (−0.6, 1.2)	0.90
Anthropometric indices						
Height-for-age z-score	−2.45 ± 0.70	−2.38 ± 0.69	0.07 (−0.05, 0.19)	−2.48 ± 0.78	−0.03 (−0.32, 0.26)	0.48
Weight-for-height z-score	−0.18 ± 0.81	−0.04 ± 0.85	0.14 (0.00, 0.28)	0.23 ± 1.00	0.41 (0.08, 0.75)	0.02
Weight-for-age z-score	−1.63 ± 0.74	−1.52 ± 0.75	0.11 (−0.02, 0.24)	−1.42 ± 0.76	0.21 (−0.09, 0.51)	0.13
rs1800629	−308GG	−308GA		−308AA		
n	477	104		3		
Age	32.1 ± 15.7	34.8 ± 15.3	2.7 (−0.6, 6.0)	47.08 ± 11.7	15.0 (−2.9, 32.8)	0.08
Sex (% male)	46.8% (223)	53.8% (56)	7% (−4%, 17%)	66.7% (2)	20% (−26%, 48%)	0.34
Haemoglobin concentration, g/L	102.3 ± 12.8	105.2 ± 12.2	2.9 (0.2, 5.6)	105.0 ± 19.2	2.7 (−11.9, 17.3)	0.10
Anaemia, % (n)^a^	69.0% (329)	64.4% (67)	−4.5% (−14.9%, 5.0%)	66.7% (2)	−2.3% (−48.4%, 25.2%)	0.54
*Plasmodium* infection, % (n)	41.1% (196)	51.9% (54)	10.8% (0.3%, 21.1%)	66.7% (2)	25.6% (−20.5%, 53.1%)	0.09
Inflammation, % (n)^b^	31.0% (148)	33.7% (35)	2.6% (−6.8%, 12.9%)	33.3% (1)	2.3% (−25.2%, 48.4%)	0.23
Mosquito net use, % (n)^c^	32.9% (157)	30.8% (32)	−2.1% (−11.3%, 8.1%)	33.3% (1)	0.4% (−27.1%, 46.5%)	0.95
Distance from homestead to dispensary, km^d^	3.6 ± 2.2	3.6 ± 2.2	0.0 (−0.5, 0.5)	3.5 ± 1.0	−0.10 (−2.6, 2.4)	1.00
Anthropometric indices						
Height-for-age z-score	−2.42 ± 0.69	−2.47 ± 0.74	−0.05 (−0.20, 0.10)	−2.69 ± 0.72	−0.27 (−1.06, 0.52)	0.61
Weight-for-height z-score	−0.11 ± 0.84	−0.17 ± 0.85	−0.06 (−0.24, 0.12)	0.07 ± 0.78	0.04 (−0.92, 1.00)	0.73
Weight-for-age z-score	−1.57 ± 0.75	−1.64 ± 0.75	−0.07 (−0.23, 0.09)	−1.56 ± 0.92	0.01 (−0.84, 0.86)	0.72

Reference/wild type allele for TNF_−1031_ is T and TNF_−308_ is G. For both genotypes the reference/wild type allele is the common allele. p-values for the associations between each factor and the allele variants investigated. Mean ± SD or % [n] unless indicated otherwise % figures represent % with feature/% without feature (number with feature/number without feature). Difference is relative to the wild type group. The difference columns are for each variable a comparison of the heterozygotes and the homozygote mutants to the wild type.

^a^Haemoglobin concentration <110 g/L.

^b^Plasma C-reactive protein concentration ≥8 mg/L.

^c^Data missing for 11 children.

^d^As the crow flies, based measurements by global positioning system.

### Associations of *TNF* genotype with malaria rates

**Figure 1:**
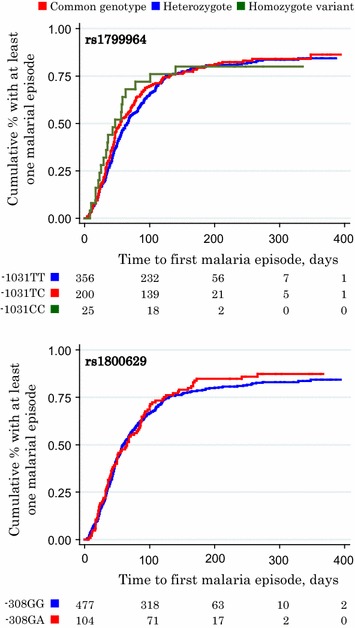
Associations of *TNF* genotype with time to first episode of malaria. Values indicate the number of children, by *TNF* genotype group, who had remained free of malaria at 0, 100, 200, 300, and 400 days of follow-up. For *TNF*_−1031_ (rs1799964) allele variant (*top panel*), pairwise group comparison with the wild type genotype as the reference group yielded p-values of 0.78 and 0.74 for heterozygotes rs1799964 TC and homozygotes (rs1799964 CC), respectively; for the *TNF*_−308_ allele variant (*bottom panel*), corresponding p-values for heterozygotes (*TNF*_−308_GA) and homozygotes (*TNF*_−308_AA) were 0.54 and 0.31, respectively (Peto tests). For homozygotes of the *TNF*_−308_A allele (*TNF*_−308_AA), the curve is not shown because only one episode occurred at 400 days of follow-up.

Overall, there were 1,511 malaria episodes recorded in 400 child-years of observation (incidence: 3.8/child-year). Of 581 children, 489 children (84%) experienced at least one malaria episode while recurrent episodes occurred in 406 (69%) children (Table 2). Compared to the wild type genotype, malaria rates (all episodes) in homozygotes for the *TNF*_−1031_C allele (*TNF*_−1031_CC) were elevated by 41% (hazard ratio 1.41; crude analysis). In the analysis of first episodes, malaria rates also seemed elevated for this genotype (hazard ratio 1.17, Table 2), but the statistical evidence for such an association was much weaker (*P* = 0.59). Only one episode of malaria occurred in homozygotes for the *TNF*_−308_A allele (*TNF*_−308_AA). This genotype was nonetheless associated with a decrease in malaria rates by 87% (hazard ratio 0.13; all episodes). Malaria rates were similarly decreased in the analysis of first episodes, but the statistical evidence was weak (*P* = 0.38). Kaplan–Meier analysis did not indicate marked group-specific differences in malaria rates for specific time periods of follow-up for either of the two *TNF* genotypes investigated (Figure 1).

**Table 2:** Associations between *TNF* genotype and rates of first and all episodes of malaria

	Homozygotes		Heterozygotes		Homozygote mutants	
rs1799964	−1031TT		−1031TC		−1031CC	
All episodes of malaria						
*n*		356		200		25
Incidence	2.93	(907/309.1)	2.92	(502/171.9)	4.15	(90/21.7)
Hazard ratio, crude	1.00	Reference	0.99	[0.85–1.14]	1.41	[1.01–1.97]
Hazard ratio, adjusted^a^	1.00	Reference	0.95	[0.82–1.09]	1.35	[1.01–1.80]
First episode of malaria						
Incidence	2.85	(296/104.0)	3.09	(168/54.4)	3.29	(20/6.07)
Incidence ratio	1.00	Reference	1.08	[0.89–1.31]	1.15	[0.68–1.76]
Hazard ratio, crude	1.00	Reference	1.08	[0.90–1.31]	1.17	[0.90–1.31]
Hazard ratio, adjusted^a^	1.00	Reference	1.07	[0.89–1.31]	1.11	[0.66–1.86]
rs1800629	−308GG		−308GA		−308AA	
All episodes of malaria						
*n*		477		104		3
Incidence	2.97	(1216/409.2)	3.08	(282/91.5)	0.36	(1/2.8)
Hazard ratio, crude	1.00	Reference	1.04	[0.87–1.24]	0.13	[0.02–0.63]
Hazard ratio, adjusted^a^	1.00	Reference	1.10	[0.93–1.29]	0.14	[0.04–0.48]
First episode of malaria						
Incidence	2.89	(392/135.6)	3.27	(91/27.8)	0.53	(1/1.9)
Incidence ratio	1.00	Reference	1.13	[0.90–1.44]	0.18	[0.00–0.98]
Hazard ratio, crude	1.00	Reference	1.07	[0.86–1.33]	0.27	[0.03–2.86]
Hazard ratio, adjusted^a^	1.00	Reference	1.14	[0.91–1.44]	0.33	[0.03–3.59]

Values between brackets indicate (episodes/child-years observed) or [95% CIs].

^a^Estimates adjusted for baseline *Plasmodium* infection status, distance between homestead and clinic (continuous variable), height-for-age z-score (continuous variable), mosquito net use and experimental intervention. There was no evidence of interactions between genotype and experimental intervention.

### Associations of *TNF* genotype with haemoglobin concentration at first malarial episode

**Figure 2:**
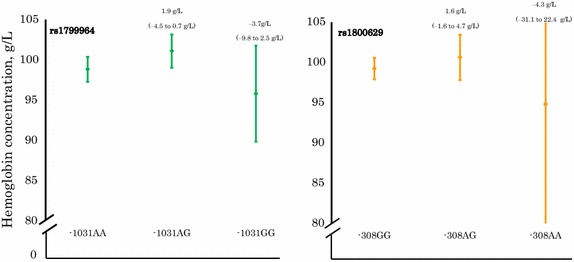
Haemoglobin concentration at first episode of malaria by *TNF* genotype. *Left panel* *TNF*_*−1031*_ (rs1799964) genotype; *right panel* *TNF*_*−308*_ (rs1800629) genotype. Genotypes are homozygous wild type, heterozygous and homozygous mutant from *left* to *right*. The data shown are means and their corresponding 95% CI. Values on *top* of *bars* indicate differences in group means with corresponding 95% CIs as obtained by multivariate regression analysis. All values are adjusted for *Plasmodium* infection status, distance between homestead and clinic, height-for-age z-score, mosquito net use, and experimental intervention. The 95% CI for haemoglobin concentration of the *TNF*_*−308*_AA genotype is outside the indicated range; this 95% CI is: 68.0–121.6 g/L.

No evidence that haemoglobin concentrations at first episode of malaria varied by variants of *TNF*_−1031_ was observed (Figure 2).

### *TNF* genotype modulation of the association between *Plasmodium* infection and haemoglobin concentration at baseline

**Figure 3:**
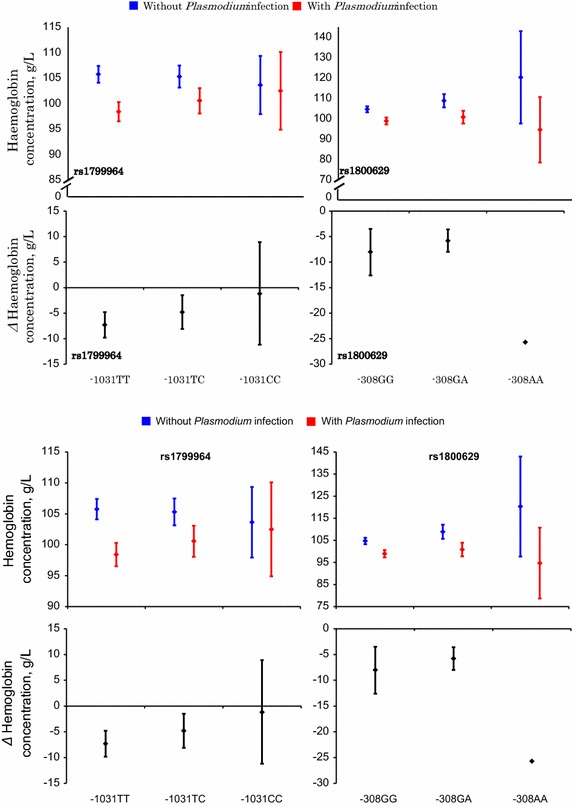
*Plasmodium* infection and haemoglobin concentrations at baseline, by *TNF* genotype. The data shown are means and their corresponding 95% CI. No confidence interval indicated for *TNF*_*−308*_AA because there was only one case of *Plasmodium* infection. All values are adjusted for age class, mosquito net use, height-for-age z-score, and distance from homestead. *Red panels* indicate haemoglobin concentration means and their corresponding 95% CI for those with *Plasmodium* infection while *blue* indicate haemoglobin concentration means and their corresponding 95% CI for those without *Plasmodium* infection. *Black panels* are for each genotype, difference in haemoglobin concentrations (means and 95% CI) between those with *Plasmodium* infection and those without *Plasmodium* infection.

No evidence that heterozygosity for either *TNF*_−1031_ or *TNF*_−308_ modified the association between *Plasmodium* infection at baseline and haemoglobin concentrations at baseline (p-values: 0.47 and 0.57, respectively) was observed. Similarly, no evidence that this association was modified by homozygosity for *TNF*_−1031_C (p-value = 0.56) was found (Figure 3).

## Discussion

In this cohort of Tanzanian children, malaria rates due to *P. falciparum* were increased in homozygotes for the *TNF*_−1031_C allele (*TNF*_−1031_CC) and decreased in homozygotes for the *TNF*_−308_A allele (*TNF*_−308_AA). No evidence that allele variants of *TNF*_−1031_ and *TNF*_−308_ were associated with haemoglobin concentration at first episode of malaria, or that they modified the association between *Plasmodium* infection and haemoglobin concentrations at baseline was found.

The associations between *TNF* allele variants and malaria rates were generally more pronounced in the analysis of all episodes than in the analysis of first episodes (Table 2). The question arises how such differences should be interpreted. Some children experience malaria more frequently than others. This between-individual variation may be due to differences in exposure to infectious mosquito bites, immunity (and thus age) and therapy-seeking behaviour. In the presence of such heterogeneity in susceptibility to malaria, ‘high risk’ individuals tend to become sick more rapidly than ‘low risk’ individuals, and once they have experienced an episode they are no longer considered in a time-to-first-event analysis. With the passing of follow-up time, only the ‘low risk’ individuals remain in the risk set and, accordingly, the event rate observed will decrease. In cohort studies such as the one described herein, this decrease is generally more pronounced in groups with high overall event rates, which have ‘high-risk’ individuals dropping out more efficiently. In a time-to-first-event analysis, group contrasts can become underestimated and associations with malaria may appear decrease with time. For this reason, the analysis of all episodes reflects with more validity associations between *TNF* allele variants and malaria rates than analysis of first episodes. In addition, the former better reflects associations with the total population burden of disease.

There was no evidence that the association between *TNF*_−1031_CC genotype and malaria rates were confounded by *Plasmodium* infection status, distance between homestead and clinic, height-for-age z-score and mosquito net use factors assessed at baseline, or by the experimental intervention. Similarly, no evidence for such confounding on the association between *TNF*_−308_AA and malaria rates was found. These associations should nonetheless be interpreted with caution, because the statistical evidence was weak in the analysis of first episodes, even though the direction of these associations was similar.

The observation that *TNF*_−1031_CC genotype was associated with increased malaria rates whilst the *TNF*_−308_AA genotype was associated with a decrease in these rates may be due to the fact that these variants tag different casual variants. This is indicated by the fact the two variants are not in linkage disequilibrium as well as no haplotype blocks were identified. A haplotype analysis previously conducted observed that many variants in *TNF* are inept markers of each other to the point that if one variant in *TNF* was a true disease susceptibility locus, most of the other variants in *TNF* would appear neutral [26]. This suggests that the disease associations with *TNF* variants are independent of each other. It may be reasonable to expect that *TNF* variants, in weak linkage disequilibrium with each other, will also be in weak linkage disequilibrium with more distant variants in the central major histocompatibility class. This is because the strength of linkage disequilibrium is determined by among others the physical proximity of two mutations and proximity in time and lineage [27]. These factors are further modified by the effects of genetic drift, migration, and natural selection [28, 29]. So it is plausible that TNF variants, while inefficient markers of each other, may be good markers of variants in more distant genes that have different disease associations.

Findings described here add to the growing but conflicting evidence that allele variants of the *TNF* gene are associated with susceptibility to malaria [9, 30, 31]. Several factors can explain apparent discrepancies in results obtained thus far in various studies. First, studies conducted have varied in design. Most studies compared hospitalized cases of severe malaria with hospital- or community-based controls [9, 30, 32, 33]. Such studies can be implemented quickly and at a relatively low cost, but they are inherently vulnerable to selection bias if the distribution of *TNF* allele variants differs between cases and controls in the absence of a true association between these allele variants and malaria. As a consequence, for studies that recruited controls from a hospital setting, the strength of the association between *TNF* allele variants and malaria will be underestimated if these controls suffered from a condition that is associated with *TNF* genotype. This is plausible, because *TNF* genotypes have also been associated with a variety of disorders other than malaria [34–37]. A better approach is to select controls that are representative of the population that produced the cases. Clark et al. [9] selected as controls cord blood samples obtained from birth clinics in the same hospital from which they recruited cases of severe malaria. Even so, bias may still have occurred in that study if *TNF* genotypes varied in frequency of delivery in hospital, or in the frequency of care-seeking behaviour and hospital admission for severe malaria. Such differentials may have occurred, for example, if the geographical distribution of *TNF* genotypes in relation to the hospital is uneven, or if *TNF* genotypes vary in the risk of birth complications. In addition, it is difficult to rule out that the results of *TNF* genotyping performed by Clark et al. in cord blood may have been affected by maternal DNA originating from contamination of cord blood at birth by maternal cells or by contact with maternal blood or tissue [38]. In this cohort study, bias may have occurred if there was differential loss to follow-up such that the risk of being lost to follow-up was related both to malaria and *TNF* genotype, or if there were group differentials in the detection of malarial episodes. However, loss to follow-up was very low (3%), genotyping was successful for almost all (95%) children, and the study is believed to have captured virtually all malaria cases [39].

Secondly, both false positives (type I errors) and false negatives (type II errors) may occur especially among studies including relatively small numbers of subjects. Indeed our study had small numbers compared to studies that have previously been conducted to investigate these kind of associations. Due to this deficiency, this study may have had reduced chance of detecting a true effect due to low power. Furthermore, the likelihood that the statistically significant results reflect a true effect may be in question. To try and reduce the possibility of sample size affecting study conclusions we have avoided other possible sources of bias by having rigorous genotyping quality measures as well as diagnosis of *P. falciparum* malaria.

Thirdly, the relationship between TNF genotype and susceptibility to malaria may depend on population-specific factors. For example, as noted in the introduction, *TNF* allele variants that are associated with an enhanced *TNF* response to chronic or repeated infections can possibly protect against newly acquired *Plasmodium* infections through an altered iron metabolism. Such protection is conceivably more effective in children who already have marginal iron status than in their iron-replete peers, and in children who are frequently exposed to infection. There may also be population-specific masking of gene variant effects (epistasis) or gene–environment interactions specific to certain populations. Additionally, haplotype heterogeneity between populations studied may exist. It has been proposed that the causal allele variants regulating *TNF* responses may actually be located downstream of the *TNF* gene and that *TNF* allele variants considered are in linkage disequilibrium with the causal variant in some populations, but not in others [9]. Much of the literature investigating the issue of whether *TNF* promoter polymorphisms have any functional effect on *TNF* transcription or influence disease susceptibility appears to report negative results, emphasizing the conclusion that polymorphisms at this locus are functionally silent and exist only due to linkage disequilibrium with selectable human leukocyte antigen (HLA) alleles. Due to this, recent studies have increasingly included more markers in the HLA classes. The relevance of HLA antigens to disease is well recognized [40, 41] and may account for many of those studies that find positive evidence of association to TNF. This emphasizes the role of TNF variants as markers for HLA or other disease loci located in the major histocompatibility class (MHC) [42, 43]. Several studies conducted within the African population have found associations between HLA alleles and *P.falciparum* malaria [44–46]. It is important to note that differences in the distribution of HLA alleles could be a cause of variation in associations between TNF polymorphisms and *P.falciparum* malaria in different geographical areas. With the increasing evidence of HLA associations with malaria, it may well be that *TNF*_−1031_ and *TNF*_−308_ are not functional variants but markers of causal variants located elsewhere.

## Conclusion

In pre-school Tanzanian children living in an area of intense transmission, the *TNF*_−1031_CC genotype was associated with increased rates of malarial episodes, whereas the *TNF*_−308_AA genotype was associated with decreased rates. Further studies are needed to confirm these findings. Additionally, further studies considering associations with the wider MHC should be conducted so as to identify candidate functional variants associated with severity of malaria.
